# 14q32.3-qter trisomic segment: a case report and literature review

**DOI:** 10.1186/s13039-016-0265-5

**Published:** 2016-08-05

**Authors:** Nicoletta Villa, Agnese Scatigno, Serena Redaelli, Donatella Conconi, Paola Cianci, Clotilde Farina, Chiara Fossati, Leda Dalprà, Silvia Maitz, Angelo Selicorni

**Affiliations:** 1 Medical Genetics Laboratory, San Gerardo Hospital, Monza, Italy; 2 Pediatric Genetic Unit, Pediatric Department of Monza Brianza per il Bambino e la sua Mamma (MBBM) Foundation, San Gerardo Hospital, Monza, Italy; 3 School of Medicine and Surgery, University of Milano-Bicocca, Milan, Italy; 4 Neonatal Intensive Care Unit, Pediatric Department at MBBM Foundation, San Gerardo Hospital, Monza, Italy

**Keywords:** Translocation (14; 21), 14q32.3-qter duplication, Array-CGH

## Abstract

**Background:**

Segmental duplication of the long arm of chromosome 14 (14q) has commonly been reported to affect the proximal segment of 14q, while distal duplication is a rare condition and often associated with segmental monosomy of other chromosomes.

**Case presentation:**

We report the clinical and genetic characterization of a 4-year-old male patient with 14q32.3-qter trisomy resulting from an adjacent segregation of a paternal reciprocal translocation (14;21)(q32.1;p12). The child shows minor facial anomalies, severe developmental delay, growth retardation, and a history of congenital hypothyroidism and neonatal transitory hyperglycemic crises.

**Conclusions:**

To the best of our knowledge, only 15 other cases of segmental 14q trisomy were documented. We compared molecularly defined cases to identify a minimal common duplicated region and to find genes with a hypothetical role in the phenotype. The presented case supports the previous suggestion of a pure “distal 14q partial duplication” and underlines the clinical variability.

## Background

Genomic rearrangements originate in the architecture of genome causing many Mendelian disorders and influencing various complex traits [1]. Sequences with a high level of homology, dispersed within and inter chromosomes, are the basis of an incorrect pairing followed by recombination; this mechanism is known as Non Allelic Homologous Recombination (NAHR). For instance, the exchange of chromosomal regions between two non homologous chromosomes, which contain paralogous repeats (also known as segmental duplications), produce a translocation.

Carriers of balanced reciprocal translocations have a high reproductive risk of conceiving chromosomally abnormal embryos, leading to recurrent pregnancy loss or birth of affected offspring [2].

The presented case (proband) is the results of a meiotic missegregation of a translocation between the 14q terminal region and a homologous sequence tract of the 21p arm (father carrier). Therefore, the child is a carrier of a triple region 14q and shows a pathological phenotype.

This abnormality has commonly been reported to affect the proximal segment of 14q, while distal duplication is a rare condition often associated with monosomic segment of other chromosomes. Only 11 cases with a pure 14q duplication are reported in the literature (Tables 1 and 2) [3–13] and only four other cases are present in Decipher database with a brief phenotypic description (Table 3; https://decipher.sanger.ac.uk/). Nine out of 16 cases (including the present one) have a molecular characterization (Fig. 3) [10–13 and 2587, 250364, 286004, 286145 from Decipher Database]. The region involved ranges from 14q31.2 to the terminal region, q32.33.

**Table 1:** Summary of clinical features from the literature review of 8 cases of distal 14q duplication (*in situ*) and present case

	Present case	Trunca et al. [3]	Orye et al. [4]	Carr et al. [5]	Masada et al. [7]	Chen et al. [10]	Thiel et al. [11]	Chen et al. [12]	Sgardioli et al. [13]
duplicated region	q32.13q32.3 paternal translocation	q31qter maternal inversion	q24q32	q31qter	32.11qter de novo duplication	q31.3q32.3 de novo duplication	q32.2qter de novo duplication	q31.3q32.12 de novo duplication	q31.3qter maternal inversion
age at diagnosis	1y 5 m	9 m	6 m	29 y	birth	45 days	8 y	PD, 6 m	20 days
patient sex	male	female	male	female	female	female	female	male	female
small at birth	+	+	-		-	-	+	-	+
MR or DD	+	+	+	+		+	+	-	+
microcephaly	+			+		+	-	-	+
hypothyroidism	+		-	-			+		+
prominent/high forehead	+	+	+		+	+	+	-	+
hypertelorism	+		+	+	-	+	+	-	+
down slanting palpebral fissures	-	-	+	-	-	+	-	-	+
broad and flat nasal bridge	+				+	-	+	-	+
bulbous nasal tip	+		+		+		+		
anteverted nares	+		+		+		-		+
dysplastic/hypoplastic ear helices	-	+	-	+	+	+	+	-	
short philtrum	-		-		-		+	-	+
thin upper lip with exaggerated Cupid’s bow	+	+	+	+	+	+	+	-	+
broad mouth	+			-	+	+	+	-	+
micrognathia	-	+	+		+	-	+	-	
brachydacytly/clinodactyly	-			digital anomalies		+	+		hypoplastic fingers
high palate	-	+	-	-	+	-			
partial agenesis/hypoplasia of corpus callosum	+		-	central cerebral atrophy		-	-	-	
congenital heart defect	+		-	-	+ ASD	+ patent ductus arteriosus	-	-	+ ASD
neural tube defect	-	-	-			-	-	-	
diaphragmatic hernia	-		-		+	-		-	+
gastroesophageal reflux disease	+			+				-	+
hypotonia	+	+	+			+		-	+
umbilical hernia	**+**		-		+	-		-	+

+: present; -: absent; *MR* mental retardation, *DD* developmental delay, *ASD* atrial septal defect

**Table 2:** Summary of clinical features of published cases of distal 14q trisomic segment derived from translocations and present case

	Present case	Mikelsaar et al. [6]	Carter et al. [8] case 4	Sutton et al. [9]
duplicated region	q32.13q32.33 21p pat	q24q32 ins(4;14)pat	q32.1qter 21p de novo	q32.3qter 22p mat
age at diagnosis	1y 5 m.	9 m	1y	3y
patient sex	male	female	male	female
small at birth	+	+		+
MR or DD	+	+	+	+
microcephaly	+			+
hypothyroidism	+	-		
prominent/high forehead	+	+		-
hypertelorism	+			+
down slanting palpebral fissures	-			
broad and flat nasal bridge	+			
bulbous nasal tip	+			-
anteverted nostrils	+			
dysplastic/hypoplastic ear helices	-			+
short philtrum	-			-
thin upper lip with exaggerated Cupid’s bow	+	+		
broad mouth	+			+
micrognathia	-	+		+
brachydacytly/clinodactyly	-			
high palate	-	+		
partial agenesis/hypoplasia of corpus callosum	+	-		+
congenital heart defect	+	-		+ VSD, ASD aortic conus
neural tube defect	-	-		+ myelomeningocele
diaphragmatic hernia	-			
gastroesophageal reflux disease	+			
hypotonia	+		+	+
umbilical hernia	+			

+: present; -: absent; *MR* mental retardation, *DD* developmental delay, *VSD* ventricular septal defect, *ASD* atrial septal defect

**Table 3:** Summary of clinical features from 4 cases of distal 14q duplication from decipher database (*https://decipher.sanger.ac.uk/* *)* and present case

	Present case	2587	250364^a^	286004	286145
duplicated region	q32.13q32.33 21p pat	q32.2q32.33 de novo	q31.2q32.33 de novo	q31.3q32.31 not reported	q32.12q32.33 de novo
age at diagnosis	1y 5 m.	not reported	1y	2y	2y
patient sex	male	not reported	female	male	female
small at birth	+	+			
MR or DD	+	+		+	+
microcephaly	+				
hypothyroidism	+				
prominent/high forehead	+	+		+	+
hypertelorism	+	+			
down slanting palpebral fissures	-				
broad and flat nasal bridge	+				
bulbous nasal tip	+				
anteverted nostrils	+				
dysplastic/hypoplastic ear helices	-				
short philtrum	**-**				
thin upper lip with exaggerated Cupid’s bow	+				
broad mouth	+	+			
micrognathia	-	+			
brachydacytly/clinodactyly	-	+			
high palate	-				+
partial agenesis/hypoplasia of corpus callosum	+		+		
congenital heart defect	+		+ ASD		
neural tube defect	**-**				
diaphragmatic hernia	-				
gastroesophageal reflux disease	+				
hypotonia	+			+	+
umbilical hernia	+				

^a^the database reports: abnormality of the face; +: present; -: absent; *MR* mental retardation, *DD* developmental delay, *ASD* atrial septal defect

The phenotype of the present case is compared with those described in literature and this allows us to identify a minimal overlapping region in 8 out of 9 cases characterized from a molecular point of view, including disease-associated genes.

Despite the rarity of distal 14q duplication, a distinctive phenotype is emerging and is characterized by low birth weight, growth retardation, psychomotor retardation, hypotonia and facial dysmorphisms.

## Case presentation

The male patient was born at 34 weeks gestation, by caesarean section in twin pregnancy (assisted reproduction, In Vitro Fertilization). Parents are apparently healthy and not consanguineous; maternal age was 38 and paternal age was 42 years at delivery. One spontaneous abortion was reported by the couple before this pregnancy. Two of the father’s sisters died during the first months of life for an unspecified heart malformation and no other information was available. Maternal family history was unremarkable. The patient’s twin sister was healthy.

The pregnancy was uneventful until 22 weeks gestation, when standard ultrasound scan showed severe intrauterine growth restriction (IUGR) of one twin, with a severe pathological doppler gradient, and oligohydramnios. Cerebellar malformation was also present; a prenatal cerebral magnetic resonance imaging (MRI) was performed but no abnormalities were detected. Fetal anatomy looked normal for gestational age.

Patient’s birth weight was 780 gr (<<3rd percentile), length 35 cm (<<3rd percentile) and head circumference 26 cm (<< 3rd percentile). Apgar score was 5 at 1st minute and 8 at 5th minute. No facial dysmorphisms were reported but short extremities and restrictive thorax were observed.

In the newborn period and in the first 12 months of life the baby suffered from various medical problems related to prematurity and oligohydramnios sequence: mild Respiratory Distress Syndrome, 1st degree bilateral intraventricular hemorrhage of 1st degree, late anemia, sepsis, osteopenia and meconium ileus (treated with ileostomy placement); he also developed parenteral nutrition-induced cholestasis. His growth was severely delayed and a gastro-esophageal reflux disease was also evident.

**Fig. 1:**
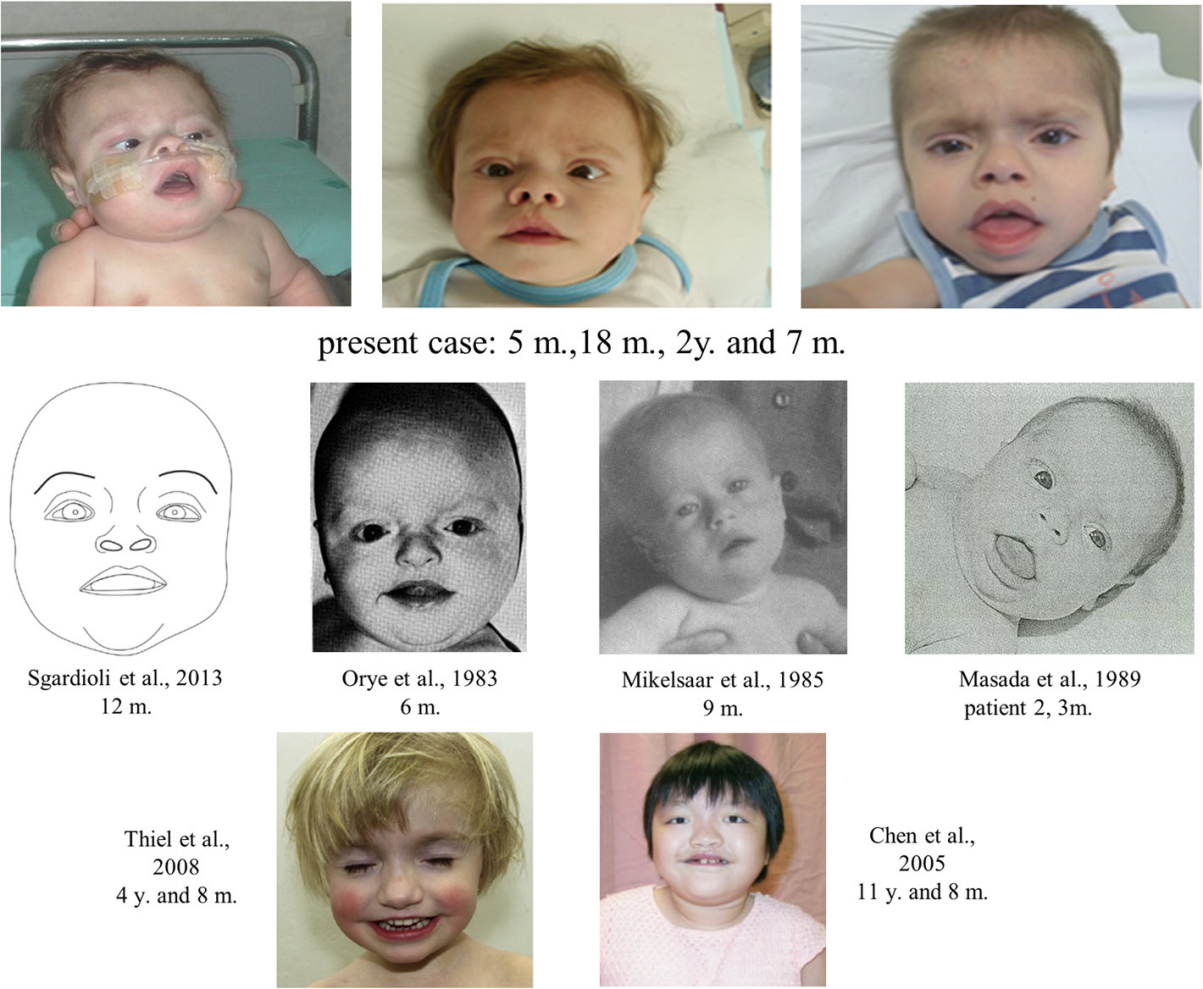
Facial features of the patient at different ages compared with literature reported faces

The child had hypoplastic kidneys with first stage chronic kidney failure and experienced hyperglycemic crises with metabolic non-ketotic acidosis during episodes of hyperthermia. Echocardiographic evaluation, performed at the age of 1 year, showed dilatation and hypertrophy of right ventricle, small apical interventricular septal defect and patent foramen ovale, right cardiac failure and secondary pulmonary hypertension. Cerebral MRI reported a thin corpus callosum, polymicrogyria, trigonal cortical heterotopia. Electroencephalography was characterized by paroxysmal record but no epilepsy crises were evident. Metabolic expansive screening and visual evoked potentials were normal. He failed the auditory brainstem response test but subsequent audiological studies were normal. Ophthalmologic evaluations showed moderate excavation of the optic disk. Hormonal studies showed a congenital central hypothyroidism with a hypoplastic thyroid gland; somatotropic hormone levels were slightly low despite adequate growth hormone levels were (IGF-1 levels <25 ng/ml with basal GH 8.6 ng/mL). Adrenal function and calcium and phosphate metabolism were normal. A negative sweat test excluded cystic fibrosis. Peroxisomal defects were also excluded on fibroblast culture. He had a nasogastric feeding tube until 15 months of age. At 18 months, his height was 60.5 cm (<<3rd percentile), weight 4.300 kg (<<3rd percentile) and head circumference 43 cm (<<3rd percentile). His face showed coarse features with frontal bossing, depressed nasal bridge with anteverted nostrils, hypoplasia of the zygomatic bones, accentuated and prominent philtrum, macrostomia, macroglossia, thick and tented upper-lip (Fig. 1). Hepatomegaly, umbilical hernia and asymmetry of lower limbs both in volume and length were also present. Neurological examination showed marked persistent axial hypotonia. At 2 years and 7 months, his height was 62.5 cm (<<3rd percentile), weight 5.150 kg (<<3rd percentile) and head circumference 44 cm (<<3rd percentile). He gained head and trunk control; axial hypotonia was important. Figure 1 reports three images of the child at different ages in comparison with faces of patients with 14q trisomic segment reported in the literature [3, 5, 6, 9, 10, 12].

At his last evaluation, the patient was 4 years and 9 months old, his height was 65.5 cm (<<3rd percentile), weight 6.680 kg (<<3rd percentile) and head circumference was 45 cm (<<3rd percentile). The language was absent, but he was able to crawl.

Prenatal diagnosis was performed on amniotic fluid sample because of IUGR and suspected cerebellar malformation identified at 22 weeks gestation in one twin. Only fetal karyotype analysis on the affected twin was done and a normal male result was obtained. At birth, uniparental disomy study for chromosomes 7 and 11 was performed, and the analysis showed biparental origin for both chromosomes (data not shown).

**Fig. 2:**
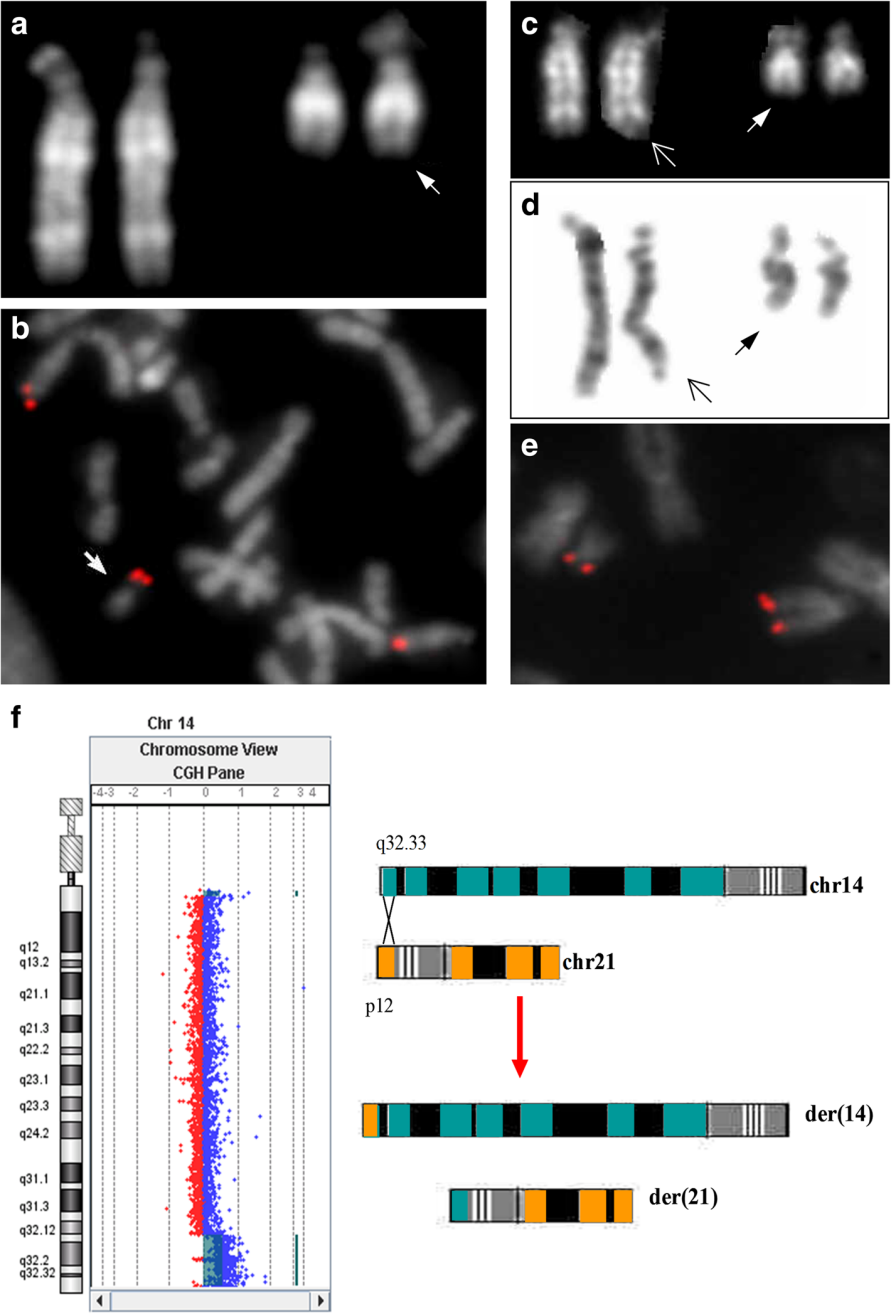
Cytogenetic, FISH and array-CGH studies. **a** Proband’s QFQ-banded chromosomes 14 and 21; the arrow shows the derivative 21. **b** FISH with subtelomeric 14q probe of the proband: the der(21) is arrowed. **c** Father’s QFQ-banded partial metaphase with two derivative chromosomes arrowed. **d** Father’s GTG-banded partial metaphase with two derivative chromosomes arrowed. **e** FISH with subtelomeric 14q probe of the father: hybridization signals are present on the normal 14 and on der(21). **f** Chromosome 14 view showing the duplication in array-CGH (*left*) and a schematic representation of supposed NAHR mechanism for translocation formation (*right*)

At 18 months of age, karyotype revaluation was required and fluorescence *in situ* hybridization (FISH) for all subtelomeric regions, performed according to the manufacturer’s specifications (Cytocell), showed normal hybridization signals for all chromosomes except for chromosome 14. Proband’s metaphases showed three hybridization signals: two at the end of the q arm of both chromosomes 14 and a third signal on the p arm of a chromosome 21, so the child was a carrier of a triple copy of 14q32.1qter region (Fig. 2a, b). Karyotype and FISH analysis of parents showed a half cryptic translocation between chromosome 14 (14q showing satellites) and 21 (p) in the father (Fig. 2c, d, e): 46,XY,t(14;21)(q32.1;p12).ish t(14;21)(DJ820M16-;DJ820M16+).

Array Comparative Genomic Hybridization (Array-CGH) analysis, performed using CGH + SNP 4x180K microarray kit (Agilent Technologies), identified a 11.44 Mb duplication on chromosome 14q arm from nt 95,849,002 (14q32.13) to nt 107,287,505 (14q32.33) in the child (genome version hg19).

The karyotype, defined following International System of Chromosome Nomenclature 2013, was: 46,XY.ish der(21)t(14;21)(q32.1;p12).ish t(14;21)(DJ820M16+).arr 14q32.13q32.33(95,849,002-107,287,505)x3 (Fig. 2f).

Uniparental disomy study for chromosomes 14 was performed, and the analysis showed biparental origin for both chromosomes (data not shown).

## Discussion

After prenatal normal karyotype result, no further study was required. The proband showed a pathologic phenotype in postnatal life, so karyotype revaluation was performed. Subtelomeric FISH and array-CGH analysis allowed to identify a trisomic portion of 14q localized on 21p arm. The paternal karyotype contained a balanced translocation which was inherited as unbalanced by the child.

A research of homology between the terminal region of chromosome 14q and the p arm of chromosome 21, through UCSC genome browser (https://genome.ucsc.edu/) and Ensemble (http://www.ensembl.org/index.html), showed a stretch of repetitive sequences of about 1.8 kb with a 96 % of homology in 14q32.33 (from nt 106,634,089 to nt 106,635,918) and 21p11.2 (from nt 10860733 to nt 10862578) with inverted orientation. Therefore a non allelic homologous recombination event, mediated by the high level of sequence homology between these two regions, could be the underlying mechanism of balanced translocation formation in the father (Fig. 2f).

To the best of our knowledge, incomplete trisomy of 14q has been reported in very few clinically documented cases [3–13]: we found a total of 11 comparable cases in the literature and other 4 cases in Decipher database (Tables 1, 2, 3 and 4). Decipher cases are molecularly well defined but lack of a detailed clinical description, follow-up and images, make difficult the comparison with literature. 8 out of 11 case reports, had distal 14q direct duplications [3–5, 7, 10–13], the remaining 3 showed translocation derivatives: the first involving a 21p arm [8], similarly to the presented case, the second a 22p arm [9] and the third an insertion into chromosome 4q [6]. Reportedly, the loss of acrocentric p arm in the translocated cases has no phenotypical consequence.

**Table 4:** Summary of clinical features from the literature review of 11 cases of distal 14q duplication and present case

	Literature	Present Case
Major Malformation		
neural tube defect	1	-
corpus callosum partial agenesis	1	+
heart defect	4	+
diaphragmatic hernia	2	-
umbilical hernia	2	+
Minor Anomalies		
prominent/high forehead	7	+
downslanted palpebral fissure	3	-
hypertelorism	6	+
dysplastic/hypoplastic ear helices	6	+
broad and/or flat nasal bridge	3	+
bulbous nasal tip	3	+
high palate	3	+
short philtrum	2	-
broad mouth	5	+
thin upper lip with exaggerated Cupid’s bow	8	+
micrognathia	6	+
digital anomalies	4	-
Medical Complications		
hypotonia	6	+
hypothyroidism	2	+
Growth and Development		
microcephaly	4	+
small at birth	5	+
developmental delay	9	+

+: present; -: absent

Our patient shows clinical features common to most types of autosomal chromosome imbalance, such as prenatal growth retardation, physical and psychomotor retardation, but minor facial dysmorphisms (high forehead, mild hypertelorism, broad nasal bridge, broad mouth), supporting the existence of a possible dysmorphic pattern caused by this trisomic 14q terminal segment (Fig. 1 and Table 4). We observed a higher prevalence of affected females (10 females versus 5 males), but it could be due to the small number of reported patients. The age at diagnosis was early, within the first year for 7 cases and earlier than 8 years for the remaining ones, with the exception of one case diagnosed at 29 years.

No particular differences were evident when unbalanced translocated (Table 2) and *in situ* duplicated patients (Table 1) were compared, indicating the absence of a positional effects.

**Fig. 3:**
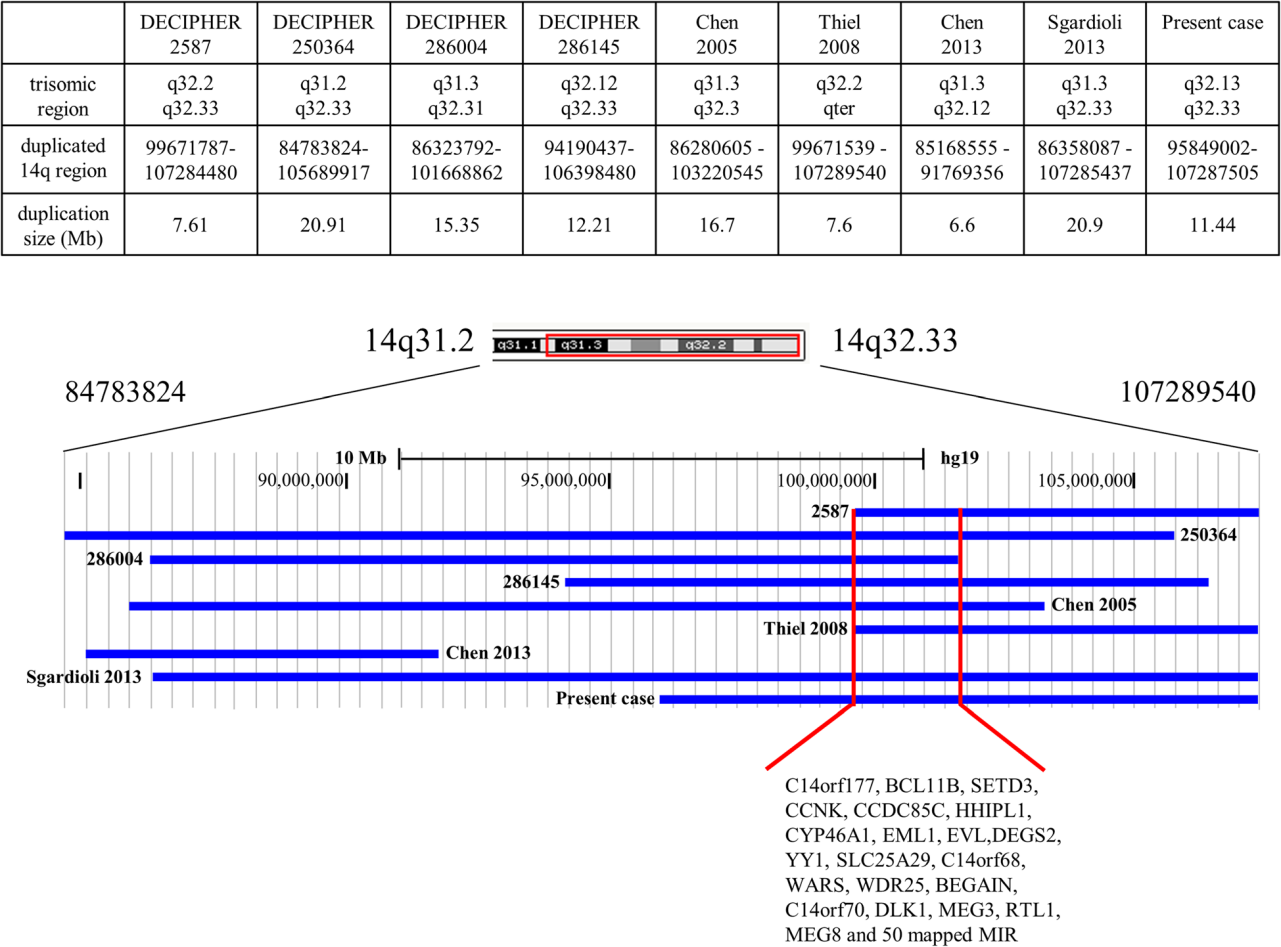
Minimal common duplicated region. Comparison of duplicated region of 9 molecularly defined cases (blue bars). All regions were converted in hg19 genome version. Vertical red bars indicate the minimal overlapping region in 8 out of 9 cases. The only case not overlapping is that described by Chen et al. [11] having a normal phenotype at 6 months of age

The minimum common region among the different cases (Fig. 3), includes a part of *DLK1-DIO3* region which contains not only paternally (*DLK1*, *RTL1*) and maternally (*MEG3*, *MEG8*) imprinted genes, but also a stretch of about 50 miRNA involved in growth and development with important regulatory functions. This is the largest cluster of miRNAs in the human genome but, to our knowledge, only miR-134 seems to be involved in mammalian brain maturation, especially in dendrite development [14]. The others appear to play roles in the onset and progression of cancers.

*DLK1* gene (OMIM 176290) is a member of Notch signalling pathway involved in cell differentiation [14], interestingly it was reported to exhibit loss of imprinting only in IUGR placentas [15]. *RTL1* (OMIM 611896) has an essential role in the maintenance of feto-maternal interface and for development of the placenta. Maternally expressed genes *MEG3* and *MEG8* (OMIM 605636 and 613648) are long non-coding RNAs with unclear function. Imprinting defect of this region cause both paternal and maternal uniparental disomies, that are characterized by two typical distinct phenotypes. The patient here reported is not affected by uniparental disomy.

Moreover, in 3 cases the gained 14q region was paternal in origin [5, 6, 9] and maternal in other 4 cases [3, 9, 11, 13]: no significant differences in the clinical features were observed underlining the lack of imprinted gene contribution in the phenotype [9]. However, the epigenetic mechanisms and the interactions among genes are not completely understood and their role in the phenotype is so far unknown.

Analysing the other genes we found that *YY1* gene participates with *SIRT1* (present in normal number of copies) at a repressor complex that normally functions to limit expression of miR134. Change in miR134 expression could result in a downregulation of *CREB* and *BDNF*, both involved in the synaptic plasticity [16].

## Conclusions

In conclusion, our observation support the existence of a “distal 14q duplication syndrome” characterized by facial dysmorphisms (high/prominent forehead, hypertelorism, downslanted palpebral fissures, wide flattened nasal bridge, broad mouth, thin upper lip with exaggerated Cupid’s bow, micrognathia), hyptonia, growth retardation and developmental delay which may be severe. Further cases will also clarify the incidence of major malformations. Here we found that congenital heart defects are the most frequent major malformations (4 out of 11 patients), while CNS anomaly and kidney hypoplasia, both observed only in our patient, seem to be rare. Furthermore, thyroid involvement will deserve specific attention in patients affected by triple copy of 14q terminal region, in order to understand its real incidence and pathophysiology.

The present case is an excellent example to argue in favour of a prenatal array-CGH study in cases with severe IUGR and normal standard karyotype and also of karyotype analysis in infertile couples especially in cases with suggestive familiar history.

## Abbreviations

Array-CGH, Array Comparative Genomic Hybridization; FISH, fluorescence *in situ* hybridization; IUGR, intrauterine growth restriction; MRI, magnetic resonance imaging; NAHR, Non Allelic Homologous Recombination
